# Associations between dental anxiety, sense of coherence, oral health-related quality of life and health behaviour – a national Swedish cross-sectional survey

**DOI:** 10.1186/s12903-015-0088-5

**Published:** 2015-09-02

**Authors:** Viktor Carlsson, Magnus Hakeberg, Ulla Wide Boman

**Affiliations:** Department of Behavioral and Community Dentistry, Institute of Odontology, The Sahlgrenska Academy, University of Gothenburg, Gothenburg, Sweden

## Abstract

**Background:**

Dental anxiety (DA) is a common condition associated with avoidance of dental care and subsequent health-related and psychosocial outcomes, in what has been described as the vicious circle of DA. Also, recent studies have found an association between the psychosocial concept of sense of coherence (SOC) and DA. More studies are needed to verify the relationship between DA and SOC, especially using population-based samples. There is also a need for studies including factors related to the vicious circle of DA, such as oral health-related quality of life (OHRQoL), in order to further establish the correlates of DA in the general population. Therefore, the aim of this study was to investigate the relationship between DA and SOC, OHRQoL and health-related behaviour in the general Swedish population.

**Methods:**

The survey included a randomly selected sample of the adult Swedish population (*N* = 3500, age 19 – 96 years.). Data was collected by means of telephone interviews. Dental anxiety was measured with a single question. The SOC measure consisted of three questions conceptualising the dimensions of the SOC: comprehensibility, manageability and meaningfulness. The data collection also included the five-item version of the Oral Health Impact Profile (OHIP-5), as a measure of OHRQoL, as well as questions on oral health-related behaviour and socioeconomic status. Statistical analyses were made with descriptive statistics and inference testing using Chi-square, *t* – test and logistic regression.

**Results:**

High DA was associated with low OHRQoL, irregular dental care and smoking. There was a statistically significant relationship between the SOC and DA in the bivariate, but not in the multivariate, analyses. Dental anxiety was not associated with oral health-related behaviour or socioeconomic status.

**Conclusions:**

This cross-sectional national survey gives support to the significant associations between high dental anxiety, avoidance of dental care and health-related outcomes, which may further reinforce the model of a vicious circle of dental anxiety. The results further indicate a weak relationship between dental anxiety and sense of coherence.

## Background

**Fig. 1:**
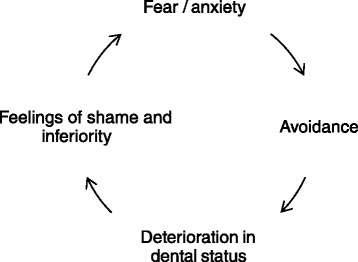
Berggren’s vicious circle of dental anxiety [6]

Dental anxiety is characterised by anxious thoughts about dentistry and fear reactions in the dental treatment situation. The two terms, dental anxiety and dental fear, are often used in the literature interchangeably to describe this psychological reaction pattern. In this paper, the term dental anxiety (DA) will be used consistently. Dental anxiety is common in the general population [1, 2] and is associated with several health-related outcomes. A strong characteristic of DA is the association with poor oral health [3–6] and avoidance of dental care [1, 7]. Several studies have also linked DA to poor oral health-related quality of life (OHRQoL) [8–13], as may be reflected by less satisfaction with facial and dental appearance [14], embarrassment related to dental status [15], pain and dysfunction [10]. Berggren, as well as other authors [16–18], have proposed a vicious circle of DA that starts with anxiety-related avoidance of dental care, followed by a subsequent deterioration in oral health and further consequences (see Fig. 1). Individuals who enter this vicious circle more often seek treatment because of an existing oral problem than visit for regular dental examinations [7], which potentially may increase the DA because of acute invasive treatments. Feelings of shame and inferiority due to poor oral health will eventually be part of the vicious circle [15–17]. Shame due to poor oral health is also included in the concept of OHRQoL. Further, it has also been suggested that general anxiety and depression may be applicable in the vicious circle [6].

A recent line of research in the DA literature concerns the sense of coherence (SOC) concept. Sense of coherence, as described by Antonovsky [19, 20], reflects a general style of perceiving and interacting with the world. It consists of three interrelated parts describing to what degree the world is perceived as comprehensible, manageable and meaningful. It is hypothesised that if a person experiences the world as coherent based on these three parts, that is, having a strong SOC, he/she would be more resilient to stress and more efficient at using coping strategies. According to Antonovsky, this would facilitate health-related behaviour and be associated with improved physical and psychological health. A relationship between the SOC and general health has also been established in the literature [21]. Sense of coherence is a salutogenic concept, which means that it is focused on factors that facilitate health and not on risk factors for disease. In line with this, the SOC has been positively associated with regular dental treatment and other oral health-related behaviour [22–24] as well as with oral health [23, 25–27] and OHRQoL [8, 13, 28]. A few studies show that a weak SOC may be associated with high DA [24, 27, 29]. From a theoretical perspective, a strong SOC may be a protective factor against DA, enabling the individual to cope with strains and anxiety related to dentistry. Individuals with a weak SOC, who do not have this protective factor, might consequently run a greater risk of developing DA.

The previously indicated relationship between DA and SOC needs further verification. In the literature, the SOC has been connected to psychological health [21], indicating a protective effect associated with a strong SOC. Dental anxiety in turn has been associated with indicators of poor psychological health [6, 30]. Additional studies investigating the salutogenic perspective in relation to DA are therefore needed, especially using population-based samples. There is also a need for studies including factors related to the vicious circle of DA, such as OHRQoL, in order to further establish the correlates of DA in the general population. Therefore, the aim of this study was to investigate the relationship between DA and the SOC, OHRQoL and health-related behaviour in the general Swedish population.

## Methods

### Subjects

The data collection was carried out as a telephone survey performed in May 2013 by TNS-SIFO, a Swedish telemarketing company that performs public opinion and market surveys. Thirty-eight questions were included in the survey, 17 of which are used in this study. The sample consisted of individuals aged 19 years or above and was randomly selected using the national SPAR registry. The SPAR registry includes all persons who are registered as residents in Sweden. Individuals with fixed or mobile phone numbers were selected. Individuals with secret phone numbers or who were unable to speak Swedish could not be included in the survey. The response rate was 49.7 %, resulting in a final study group of 3 500 individuals. The study was approved by the Regional Ethical Review Board in Gothenburg, reg. no 801–12. Each individual asked to participate was informed about the study according to the Swedish Research Ethical law and regulations, and informed consent was achieved by means of verbal approval to participate.

### Instruments

*Dental anxiety* was measured with the Dental Anxiety Question (DAQ) [31], a single-item measure asking the respondent if he/she is anxious about going to the dentist. The response alternatives were: “no”; “a little”; “yes, quite”; or, “yes, very”. Dental anxiety has often been measured with single questions in previous research and the DAQ has shown good validity [31]. For analysis, DA was dichotomised into low DA (“no” and “a little”) and high DA (“yes, quite” and “yes, very”). Furthermore, DA was also analysed with regard to the most extreme outcomes; i.e., no DA (response option “no”) and extreme DA (response option “yes, very”), respectively.

*Sense of coherence* was measured using a three-item scale (SOC-3), where each item is operationalised to measure one of the three SOC dimensions: manageability, meaningfulness and comprehensibility [32]. The questions used for each item were, for manageability, ‘Do you usually see a solution to problems and difficulties that other people find hopeless?’; for meaningfulness, ‘Do you usually feel that your daily life is a source of personal satisfaction?’, and for comprehensibility, ‘Do you usually feel that the things that happen to you in your daily life are hard to understand?’. Each item was given a score between zero and two based on the response alternatives, “Yes, usually”, “Yes, sometimes”, and “No”. Reversed scoring was used for comprehensibility compared with the other two items. A composite score was calculated ranging from zero to six, where a higher score indicates a lower SOC level. Lundberg and Peck [32] reported acceptable reliability for the scale. The scale has been used in a substantial amount of research and has been compared to Antonovsky’s original 29-item measure of SOC, showing a correlation of *r* = 0.72 between the two measures [33]. Following previous research [34, 35], the total score of the scale was dichotomised for analysis using a score of zero to two to indicate a strong SOC and a score of three to six to indicate a weak SOC.

*Oral health-related quality of life* was measured using a five-item version of the Oral Health Impact Profile (OHIP-5) [36, 37]. The Swedish version of the OHIP-5 has shown acceptable internal reliability with a Cronbach’s alpha of 0.77, and is highly correlated (*r* = .92) with the Swedish version of the original 49-item scale [37]. The five items ask about the presence of functional limitation, physical pain, psychological discomfort, physical disability and handicap. The questions are answered on a five-point Likert scale from 1 = “never” to 5 = “very often”, and a total score of 5–25 is calculated, where a higher score indicates lower OHRQoL. In this study, the OHIP-5 was dichotomised for analysis. In previous research, the 14-item versions of the OHIP scale have been scored according to the number of items that indicate an existing problem; i.e., the total number of items with a score of three or higher [8, 28]. Following this method, it was decided to make the dichotomization between individuals who scored three or higher on no or one item (high OHRQoL) and individuals who scored three or higher on two to five items (low OHRQoL).

#### Oral and general health-related behaviour

Tooth-brushing and flossing frequency were measured separately, with the response alternatives of: ≥ 3 times a day; twice a day; once a day; several times a week; once a week; seldom or never. For the analysis, tooth-brushing was dichotomised into ≥ twice a day, or ≤ once a day. Flossing was dichotomised into ≥ once a week, or seldom or never. Attendance patterns were measured with a question measuring the frequency of dental care visits as: twice a year; once a year, once every other year; more seldom than every other year; only acutely; or, never. For the analysis, dental care attendance was split into “regular” (yearly and once every other year) or “irregular” (less often than every other year, only acutely or never) dental care. Smoking was measured with the question “Do you smoke on a daily basis?” and the response options: “Yes”; “No, but have smoked before”; “No, do not smoke/have never smoked”. For the analysis, smoking was dichotomised into “daily smokers” and “not daily smokers”.

*Level of education* was measured with the options: ≤ 9 years (low); 10–12 years (medium); > 12 years [high; (studies at college or university; degree from college or university; PhD)]. *Country of birth* was measured with the options of: Sweden; another Nordic country; non-Nordic country.

### Statistics

Statistical analyses were made with descriptive statistics and inference testing using Chi-square, *t* – test and logistic regression. All calculations were made using the SPSS version 19.0 (IBM Corp, Armonk, NY, USA), except for comparisons with data from Statistics Sweden (SCB), which were calculated using Excel 2010 (Microsoft Corp.).

The chosen level of significance was *p* < 0.05. Of the total study group, 245 individuals had one or more missing items on the SOC-3 scale, and these individuals were excluded from the analyses regarding SOC. Thirty-seven individuals had one or more items missing on the OHIP-5 scale, and they were likewise excluded from the analysis regarding OHRQoL. Further, some items were missing on most of the remaining measures and, thus, the number of excluded individuals for these measure were: age, five individuals; DA, two individuals; smoking, four individuals; country of birth, three individuals; education, 22 individuals; tooth flossing, 11 individuals, and regularity of dental care, three individuals.

## Results

### Characteristics of the study group

The total sample consisted of 3500 individuals (53.1 % female and 46.9 % male). The mean age was 53.4 years (*SD* =17.5, range 19–96 years). Characteristics of the study group are reported in Table 1.

**Table 1:** Characteristics of the study group in the total sample and divided by level of dental anxiety (DA)

Variable	Total group	Low DA	High DA
	(*N* = 3500)	(*n* = 3175)	(*n* = 323)
Sex			
Men/Women	46.9 %/53.1 %	48.8 %/51.2 %*	28.5 %/71.5 %*
Age (years)	53.4 (17.5)	53.9 (17.7)*	49.3 (15.0)*
Education			
Low	18.1 %	18.2 %	16.5 %
Medium	40.2 %	39.7 %	45.2 %
High	41.7 %	42.1 %	38.3 %
Country of birth			
Sweden	89.2 %	89.4 %	87.0 %
Another Nordic country	2.8 %	2.7 %	3.1 %
Non-Nordic country	8.1 %	7.9 %	9.9 %
SOC			
Strong/Weak	89.3 %/10.7 %	89.7 %/10.3 %	86.2 %/13.8 %
OHRQoL			
High/Low	90.8 %/9.2 %	92.3 %/7.7 %*	75.7 %/24.3 %*
Dental care			
Regular/Irregular	90.6 %/9.4 %	91.7 %/8.3 %*	80.2 %/19.8 %*
Daily tooth-brushing			
≥ twice/≤ once	93.2 %/6.8 %	93.0 %/7.0 %	95.4 %/4.6 %
Dental floss frequency			
≥ weakly/more seldom	52.9 %/47.1 %	53.2 %/46.8 %	50.9 %/49.1 %
Daily smoking			
Yes/No	9.1 %/90.9 %	7.9 %/92.1 %*	20.5 %/79.5 %*

All numbers are presented as percentages or means (SD)

*p < .001, statistical tests were performed in order to compare the high and low DA groups

### Participation analysis

Comparisons between the study sample and data from 2013 for the total Swedish population aged 19 or older (hereafter termed “the general Swedish population”) were made to investigate the representativeness of the study sample. Data for the general Swedish population were obtained from Statistics Sweden (SCB, www.scb.se). In our sample, 10.8 % stated that they were born in a country other than Sweden, which differs from the general Swedish population where 18.0 % are foreign-born (*χ*^2^ = 122.63, *p* < .001). There was also a gender difference in that our sample included somewhat more women than the general Swedish population (53.1 % vs. 50.5 %, *χ*^2^ = 9.58, *p* < .01). There was a smaller proportion of individuals with low and medium-high education, and a higher proportion of highly educated individuals in our sample, compared with the general Swedish population (low 18.1 % vs. 19.7 %, medium 40.2 % vs. 45.8 %, and high 41.7 % vs. 34.5 %, *χ*^2^ = 80.19, *p* < .001). The mean age in our sample was 53.4 years, while it was approximately 49.4 years in the general Swedish population.

### Dental anxiety

Of those included in the study group, 9.2 % were classified as belonging to the high DA group and 90.8 % to the low DA group. There was a gender as well as an age difference between the high and low DA groups (see Table 1). Individuals in the high DA group were more often female (*χ*^2^ = 48.53, *p* < .001) and younger (*t* = 5.16, *p* < .001). No statistically significant differences in education and country of birth were found between the high and low DA groups. When analysing the most extreme outcomes, 81.0 % of the sample stated no DA and 4.7 % of the sample stated extreme DA.

### Sense of coherence

The mean score in the study group on SOC-3 was 1.1 (*SD* = 1.1, *MD* = 1, range 0–6), and women and men did not differ with regard to mean or median score [*M* = 1.1 (*SD* = 1.1) and *MD* = 1 for both women and men]. In the sample, 10.7 % were classified as having a weak SOC based on SOC-3 scores (see Table 1). There was no statistically significant difference between men and women in the prevalence of a weak SOC. Because of the number of individuals with missing values on the SOC-3 (*n* = 245) non-response analyses for the SOC-3 were performed regarding age and gender. The results revealed a higher mean age among individuals with missing items on the SOC-3 compared to the remainder of the sample [*M* = 62.5 years (*SD* = 18.5) vs. *M* = 52.8 years (*SD* = 17.2), *t* = 8.4, *p* < .001]. No statistically significant difference was found concerning gender.

### Sense of coherence and dental anxiety

No statistically significant difference concerning the proportion of individuals with a weak SOC was found between the high and low DA groups (low DA group: 10.3 % vs. high DA group 13.8 %, *χ*^2^ = 3.49, *p* = .062, see Table 1). To further explore the relationship between DA and a weak SOC, DA was analysed regarding its most extreme outcomes; i.e., no DA and extreme DA, respectively. In individuals with no DA, 9.5 % had a weak SOC compared with 16.2 % in individuals with extreme DA resulting in a statistically significant difference (*χ*^2^ = 7.30, *p* = .007).

### Oral health-related quality of life

The mean score on the OHIP-5 was 6.8 (*SD* = 2.2, range 5–23), with a median of 6 for the total study population. Women scored slightly higher than men [*M* = 7.0 (*SD* = 2.3) and *M* = 6.6 (*SD* = 2.0), respectively, *t* = − 4.8, *p* < .001)], which indicates somewhat lower OHRQoL for women compared with men. Of the total sample, 9.2 % were classified as having low OHRQoL based on OHIP-5 scores (see Table 1).

### Oral health-related quality of life and dental anxiety

A relationship between OHRQoL and DA was found when the high and low DA groups were compared. The prevalence of low OHRQoL was 7.7 % in the low DA group and 24.3 % in the high DA group (*χ*^2^ = 95.55, *p* < .001, see Table 1).

### Health-related behaviour and dental anxiety

The prevalence of health-related behaviour in the total study group, and divided by high and low DA, is reported in Table 1. Statistically significant differences between the high and low DA groups were found, which reflected more irregular dental attendance (*χ*^2^ = 45.52, *p* < .001) and daily smoking (*χ*^2^ = 56.11, *p* < .001) in the high DA group (see Table 1).

### Regression analysis

Based on theoretical considerations and differences in the bivariate analyses, a logistic regression analysis was performed with the level of DA as the dependent variable. The independent variables were age, smoking, gender, dental attendance patterns, OHRQoL and SOC (see Table 2). In the regression analysis, a low SOC was not associated with high DA. Low OHRQoL, female gender, irregular dental care and smoking were strongly associated with high DA. Increasing age had a statistically significant relationship with low DA. To further explore these relationships, a new logistic regression analysis was performed with DA as the dependent variable, but this time it was analysed with regard to its most extreme outcomes; i.e., no DA and extreme DA, respectively. The independent variables remained the same as in the first logistic regression analysis. All variables were significant predictors of DA except for the SOC (*p* = .081, see Table 3).

**Table 2:** Multivariate logistic regression (*N* = 3214) with dental anxiety (DA) as the dependent variable (low DA = 0; high DA = 1)

Independent variable	OR	95 % CI	*p*
Female gender	2.40	1.84 – 3.15	< .001
Age (years)	0.99	0.98 – 0.99	< .001
Low SOC	1.16	0.80 – 1.68	.437
Low OHRQoL	2.99	2.19 – 4.09	< .001
Irregular dental care	2.27	1.62 – 3.17	< .001
Daily smoking	2.68	1.93 – 3.71	< .001

Independent variables: gender, age, sense of coherence (SOC), oral health-related quality of life (OHRQoL), regularity of dental care and smoking

**Table 3:** Multivariate logistic regression (*N* = 2742) with the dental anxiety (DA) extreme group as the dependent variable (no DA = 0; extreme DA = 1)

Independent variable	OR	95 % CI	*p*
Female gender	3.43	2.31 – 5.10	< .001
Age (years)	0.99	0.98 – 1.00	.015
Low SOC	1.54	0.95 – 2.51	.081
Low OHRQoL	2.67	1.73 – 4.13	< .001
Irregular dental care	3.75	2.46 – 5.70	< .001
Daily smoking	3.98	2.65 – 5.98	< .001

Independent variables: gender, age, sense of coherence (SOC), oral health-related quality of life (OHRQoL), regularity of dental care and smoking

## Discussion

In this cross-sectional national survey, high DA was related to low OHRQoL, irregular dental attendance patterns, smoking, age and gender. These variables strongly predicted high DA in the logistic regression analysis. In the bivariate analyses, the relationship between DA and SOC was statistically significant when DA was analysed with regard to its most extreme outcomes; i.e., no DA vs. extreme DA. In that analysis, extreme DA was associated with a weak SOC. Moreover, the multivariate model with the most extreme DA outcomes indicated a moderate Odds Ratio, a 54 % increased likelihood of being extremely dentally anxious when reporting a low SOC value, albeit a non-significant result.

In accordance with the present study, SOC has been related to high DA in previous research [24, 27, 29]. As a comparison to this study, who found a weak relationship between SOC and DA, a much stronger relationship was found by Jaakkola *et al.* [29]. There may be a couple of reasons for why these results differ. Firstly, there were differences in the populations studied. In the Jaakkola *et al.* study, only 18-year-old adolescents where included, while the present study included adults aged 19–96 years old. The sense of coherence is believed to stabilise in the mid-twenties and DA has been found to decrease with higher age, which may yield a more dentally anxious group with a more unstable SOC level in the Jaakkola *et al.* study [29]. Secondly, there were differences in measurements and analyses. The present study used a simplified measure of SOC, which may not be as precise as the 13-item version of Antonovsky’s Sense of Coherence Questionnaire (SOC-13) used by Jaakkola *et al.* Further, no established cut-off of the SOC-13 has been presented and the chosen median as a cut-off may have influenced the results in the Jaakkola *et al.* study. Also, there was a difference regarding the instruments used to measure DA, where Jaakkola *et al.* included the Modified Dental Anxiety Scale, which is a more elaborate measure than the one used in this study. Few independent variables were included in the logistic regression analysis by Jaakkola *et al.*, which may have strengthened the SOC results in that study.

The precise nature and strength of the relationship between the SOC and DA remains to be clarified. It is possible that it varies with contextual factors and populations, although all of the previously mentioned studies, including this one, point to some relationship between the SOC and DA. Theoretically, a strong SOC should be protective, in that it could prevent individuals from developing stress when dealing with the strains of life. In that way, the SOC may be a protective factor against the development of DA and, therefore, following the theory, high DA ought to be associated with a weaker SOC. When stress occurs, which it inevitably does in most of us from time to time, regardless of the SOC level, a high SOC is hypothesised to be protective in that it strengthens appropriate coping behaviour. In this way, a strong SOC may be related to a greater ability to endure dental care in individuals with high DA and, as a consequence, maintain good oral health and a high level of OHRQoL. Further research is needed to establish the existence and nature of these relationships.

The SOC-3 scale used in this study employs a different approach than Antonovsky’s SOC-13 scale, in that it tries to capture the most essential parts of the SOC concept in three focused questions. It may be important to try to capture the same phenomena with different measures, as this might provide a broader and more reliable picture. However, it also makes comparisons between studies more difficult. Further, it is essential in epidemiological research that questionnaires are kept short to make data collection possible in large samples. The proportion of individuals with a weak SOC in this study was low, compared with Swedish general population data collected by Lundberg & Nyström Peck in 1991, using the same measure [32] (10.7 % vs. 19.0 %). These differences may be due to societal changes in Sweden during the past 20+ years.

In this study, the five-item version of the OHIP scale was used to measure OHRQoL. The median value of the OHIP-5 was the same as the 50th percentile in Swedish normative data reported by Larsson *et al.* [37], indicating an expected general level of OHRQoL. The finding that high DA was associated with low OHRQoL was expected and is well in line with previous research [8–13]. The results provide further evidence of the association between high DA and functional and psychosocial consequences. In the vicious circle model of DA, some authors have focused on feelings of shame and inferiority and how these feelings further increase the DA [15–17]. The full range of OHRQoL consequences of DA seems to be broader, not limited to feelings of shame and inferiority and, as previously mentioned, general categories of mental health has been suggested to be applicable within the model [6]. This may set the stage for a more comprehensive OHRQoL perspective to be included in the vicious circle of DA; however, it remains to be investigated whether impaired OHRQoL, as conceptualised here as consequences, also reinforces DA. If that turns out to be the case, OHRQoL may deserve a prominent position in the model.

The current study also found a previously reported relationship between DA and smoking [38, 39]. Besides being a risk factor for poor oral health, smoking may be seen as a surrogate measure of health behaviour that is external to the vicious circle model of DA. It may be that smoking is indicative of several potentially negative and perhaps mediating factors in the vicious circle of DA. This is reflected by results associating smoking with decreased dental attendance, both in individuals with DA [40] and in the general population [41, 42]. Tooth-brushing or flossing was not related to DA in this study, although some previous research has indicated a relationship between DA and oral hygiene behaviour [43, 44]. The variation in tooth-brushing frequency was small with 93 % of the sample stating that they brushed twice a day or more often. It is common knowledge in Sweden that the teeth should be brushed at least twice a day, which may make responders more susceptible to give a socially acceptable answer to this particular question, which in turn may have influenced the results. The generally high knowledge concerning oral hygiene behaviour in Swedish adults may also explain why there were no major differences regarding this aspect in relation to DA.

The strengths of this cross-sectional study were the large random national sample and the use of established scales. The study included several measures relevant to DA, including parts of the vicious circle of DA and factors external to it. Short scales consisting of one to five questions were used to capture the different concepts investigated. These measures may not be as precise as more elaborate measures, which may be considered as a weakness. Missing items on the SOC-3 scale was associated with older age, which may indicate difficulties in answering the SOC-3 questions among older individuals. Another weakness in the current study was the response rate of 49.7 %. According to comparisons with statistics for the total population of Sweden aged 19 or older, the sample in this study was somewhat better educated, older, less often born outside of Sweden and consisted of proportionally more women. These discrepancies may have influenced the results to some degree but, at the same time, the study sample did include a wide range of individuals, as indicated by age, level of education and county of birth.

## Conclusions

This large national survey, performed on a representative adult sample, found that low OHRQoL, irregular dental care, female gender and smoking all predicted high DA. The study also found a relationship between the SOC and DA, although this relationship was weaker. This study gives further support to the associations between high DA, avoidance of dental care, and health-related outcomes, which may further reinforce the model of a vicious circle of DA.

## Abbreviations

DA: Dental anxiety
DAQ: Dental anxiety question
OHIP-5: Oral health impact profile, five-item version
OHRQoL: Oral health-related quality of life
SCB: Statistics Sweden
SOC: Sense of coherence
SOC-3: Concept of sense of coherence, three-item scale
